# A bacteria-specific 2[4Fe-4S] ferredoxin is essential in *Pseudomonas aeruginosa*

**DOI:** 10.1186/1471-2180-10-271

**Published:** 2010-10-28

**Authors:** Sylvie Elsen, Georgios Efthymiou, Panagiotis Peteinatos, George Diallinas, Panayotis Kyritsis, Jean-Marc Moulis

**Affiliations:** 1 Laboratoire de Biochimie et Biophysique des Systèmes Intégrés; iRTSV, CEA, Grenoble, France; 2 BBSI, CNRS, Grenoble, France; 3 Université Joseph Fourier, Grenoble, France; 4 Department of Inorganic Chemistry, Faculty of Chemistry, University of Athens, Panepistimioupolis, Athens 15771, Greece; 5 Department of Botany, Faculty of Biology, University of Athens, Panepistimioupolis, Athens 15781, Greece; 6 Laboratoire de Chimie et Biologie des Métaux, iRTSV, CEA, Grenoble, France; 7 LCBM, CNRS, Grenoble, France

## Abstract

**Background:**

Ferredoxins are small iron-sulfur proteins belonging to all domains of life. A sub-group binds two [4Fe-4S] clusters with unequal and extremely low values of the reduction potentials. These unusual properties are associated with two specific fragments of sequence. The functional importance of the very low potential ferredoxins is unknown.

**Results:**

A bioinformatic screening of the sequence features defining very low potential 2[4Fe-4S] ferredoxins has revealed the almost exclusive presence of the corresponding *fdx *gene in the *Proteobacteria *phylum, without occurrence in *Archaea *and *Eukaryota*. The transcript was found to be monocistronic in *Pseudomonas aeruginosa*, and not part of an operon in most bacteria. Only *fdx *genes of bacteria which anaerobically degrade aromatic compounds belong to operons. As this pathway is not present in all bacteria having very low potential 2[4Fe-4S] ferredoxins, these proteins cannot exclusively be reductants of benzoyl CoA reductases. Expression of the ferredoxin gene did not change in response to varying growth conditions, including upon macrophage infection or aerobic growth with 4-hydroxy benzoate as carbon source. However, it increased along the growth curve in *Pseudomonas aeruginosa *and in *Escherichia coli*. The sequence immediately 5' upstream of the coding sequence contributed to the promotor activity. Deleting the *fdx *gene in *Pseudomonas aeruginosa *abolished growth, unless a plasmid copy of the gene was provided to the deleted strain.

**Conclusions:**

The gene of the very low potential 2[4Fe-4S] ferredoxin displays characteristics of a housekeeping gene, and it belongs to the minority of genes that are essential in *Pseudomonas aeruginosa*. These data identify a new potential antimicrobial target in this and other pathogenic *Proteobacteria*.

## Background

Ferredoxin (Fdx) is the name given to a variety of small proteins binding inorganic clusters organized around two to four iron atoms and a complementary number of sulfur atoms [1]. Complete genomic sequences have revealed the presence of a very large number of genes encoding such proteins, mainly in bacteria and archaea [2].

Fdxs are most often assigned electron transfer roles and some of them occupy central positions in metabolism [3], but the roles of a majority of Fdxs remain unknown [4,5]. Functional substitution among Fdxs may occur, and other soluble electron shuttles, such as flavodoxins, may act as Fdx-substitutes. This is the case upon iron starvation for a 2[4Fe-4S] Fdx in glycolytic *Clostridia *[6] or a [2Fe-2S] Fdx in some photosynthetic organisms [7], for instance. Despite this apparent functional redundancy, most sequenced genomes display a wealth of genes encoding various Fdxs. For example, the reference PAO1 strain of the opportunistic pathogen *Pseudomonas aeruginosa *[8] has at least 6 genes encoding Fdxs of different families. A flavodoxin (PA3435) is also present in this strain. It is often unclear in which reactions Fdxs are involved and which biological function relies on a given Fdx.

**Figure 1:**
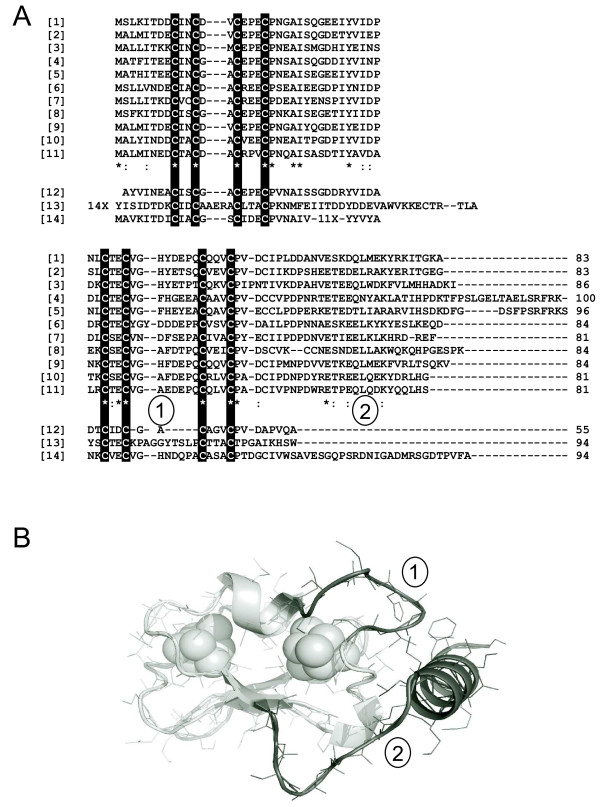
**Characteristic features of Fdx of the AlvinFdx family**. (A) Sequence alignment of selected 2[4Fe-4S] Fdxs from γ-proteobacteria [1]*Pseudomonas aeruginosa *PAO1, [2]*Allochromatium vinosum *DSM180, [3]*Escherichia coli *K12-MG1655; δ-proteobacteria [4]*Anaeromyxobacter dehalogenans *2CP-C, [5]*Plesiocystis pacifica *SIR-1; ε-proteobacteria [6]*Helicobacter pylori *26695, [7]*Campylobacter jejuni *NCTC 11168, Cj0354 sequence; Chloroflexi [8]*Dehalococcoides sp. VS*; β-proteobacteria [9]*Azoarcus sp*. (or *Aromatoleum aromaticum*) EbN1 (locus NT01AE0820), [10]*Thauera aromatica *K172; α-proteobacteria [11]*Rhodopseudomonas palustris *CGA009; [12]*Clostridium acidurici *as an example of heterotrophic anaerobic bacteria; [13]*Azoarcus sp*. EbN1 (locus NT01AE3314) belonging to the *bcr *cluster; [14]*Campylobacter jejuni *NCTC 11168 Cj0333 sequence. *n*X stands for insertions of *n *aminoacids. Stars on the consensus line for proteins of the AlvinFdx family indicate identical residues and colons are for conserved residues. The ① and ② symbols lie under non-conserved residues belonging to the fragment between cysteine ligands and the turn and helix addition, respectively, that characterize the AlvinFdx family as indicated in the structure of Figure 1B. The lengths of the compared sequences are given at the end of the alignment, and [4Fe-4S] cysteine ligands are boxed. (B) View of the *P. aeruginosa *Fdx structure [10]. The general fold is shown (light grey tube) with the 8 amino acid stretch between two cysteine ligands of one cluster (labelled ①) and the turn and helix at the C-terminus ② colored in dark grey. Iron and inorganic sulfur atoms are represented as spheres.

One of *P. aeruginosa *Fdxs is encoded by the PA0362 locus (*fdx1*) and it belongs to a separated family of proteins containing two [4Fe-4S] clusters [9]. The sequences of proteins of this family are characterized by a segment of six amino acids between two cysteine ligands of one cluster and a C terminal extension of more than 20 amino acids beyond the last ligand of the other cluster (Figure 1). The structure of this Fdx [10] shows that these two characteristic peptides fold as a particular turn close to one cluster and as a turn and a long α-helix, respectively (Figure 1). The first member of this family (hereafter abbreviated AlvinFdx) to be identified was that of the purple sulfur γ-proteobacterium *Allochromatium vinosum*, originally named *Chromatium vinosum*, and it was initially classified among other [4Fe 4S] 'bacterial' Fdxs (as opposed to 'plant' [2Fe 2S] Fdxs) [11]. It was later found that the characteristic sequence differences of proteins of the AlvinFdx family shifted the reduction potential of the [4Fe 4S] clusters to very negative values, below -450 mV with reference to the Normal Hydrogen Electrode, with one reaching -650 mV or less [12]. Because of this unusual property, it is not easy to find an efficient physiological reductant for such proteins, especially in non-photosynthetic organisms. Additional unique spectroscopic [13] and structural [10,14,15] properties have also been evidenced in these proteins.

A well defined function for members of this family of Fdxs has only been found in bacteria catabolizing aromatic compounds in the absence of oxygen [16]. The *Thauera aromatica *Fdx participates in an electron transfer chain, as electron acceptor from 2-oxoglutarate:Fdx oxidoreductase and donor to benzoyl-CoA reductase [17]. However, this particular Fdx displays the less negative reduction potentials among Fdxs of the AlvinFdx family [18], and this catabolic pathway is not always present in all bacteria containing *fdx *genes (i.e. genes encoding Fdxs similar to that of AlvinFdx).

Since the role of Fdxs of the AlvinFdx family is not known in most bacteria (those that do not anaerobically catabolize aromatics), the importance of the *fdx1 *gene of the *P. aeruginosa *PA0362 locus has been investigated in the present work. The possibility of endogenous *in vivo *functional substitution has been examined by removing the chromosomal copy of the gene. Also, the main properties of *fdx1 *expression have been explored and the distribution of similar genes has been analyzed in the available sequence databases. These newly obtained data strongly indicate a non-exchangeable and housekeeping function for *fdx1*.

## Results

### In silico inventory of genes encoding AlvinFdx

The signature of AlvinFdx sequences encompasses two components. First, a 6-7 amino acids insertion separates two iron-coordinating cysteines of one cluster, whereas [4Fe-4S] clusters in Fdxs are usually bound by a stretch of three cysteines, two residues apart in the sequence. Second, a 27-43 amino acids fragment, following the last coordinating cysteine at the C-terminus, partly folds as an α-helix (Figure 1). Over the last 15 years, extensive genome sequencing has revealed numerous *fdx *genes encoding protein sequences with characteristics of the AlvinFdx family, but no systematic inventory has been carried out. Peptidic insertions may change the properties of proteins in unpredictable ways, as exemplified by the large differences between the Fdxs studied here and more conventional, shorter (ca. 55 amino acids) 2 [4Fe-4S] ones [12,13,15]. Therefore, the present analysis is restricted to proteins of no more than 100 amino acids showing the above two sequence features.

Genes encoding proteins with the above characteristics in the sequence databanks were only found in the (eu)bacterial domain: more than 200 such genes were detected. Although *Archaea *are a very abundant source of iron-sulfur proteins, no genes encoding proteins of the AlvinFdx family, as precisely defined above, could be identified in this domain. Within bacteria, the occurrence of *fdx *genes was restricted to *Chloroflexi *(in only the *Dehalococcoides *genus), to *Nitrospirae *(in only the *Leptospirillum *genus), and to the *Proteobacteria*. In the latter phylum, all α to ε classes were represented (Figure 1A), but with noteworthy differences. All fully sequenced species of β- and ε- *Proteobacteria *displayed the *fdx *gene, which was also present in a large number of, but not all, γ-*Proteobacteria*. In contrast, the *fdx *gene was found in only a minority of the δ-*Proteobacteria *genera (*Anaeromyxobacter, Plesiocystis, Sorangium*), and in only one species, *Rhodopseudomonas palustris*, of α-*Proteobacteria*.

### Genomic organization of Alvin-like Fdx genes

**Figure 2:**
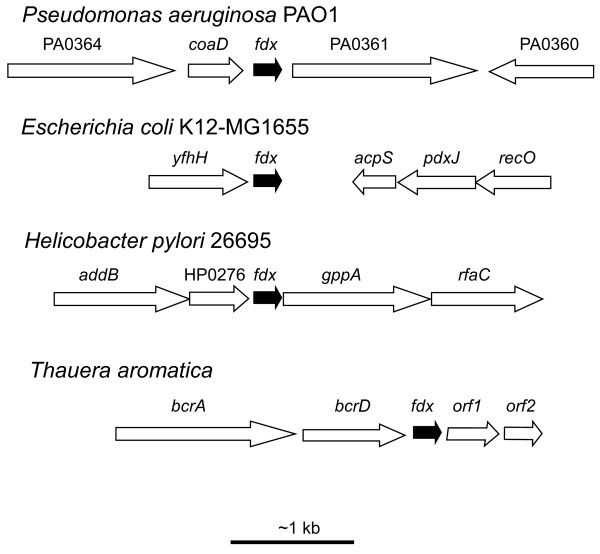
**Genomic context around genes of the AlvinFdx family in selected bacteria**. The predicted ORFs neighbouring *fdx *are approximately drawn to scale (shown at the bottom) with arrows indicating the direction of transcription. Genes and encoded proteins: *P. aeruginosa *PAO1: PA0364, probable oxidoreductase; *coaD*, phosphopantetheine adenylyltransferase; PA0361, probable γ-glutamyltranspeptidase precursor; PA0360, conserved hypothetical protein. *E. coli *K12-MG1655: *yfhH*, conserved hypothetical protein; *acpS*, CoA:apo-[acyl-carrier-protein] pantetheinephosphotransferase; *pdxJ*, pyridoxin 5'-phosphate synthase; *recO*, protein that interacts with RecR and possibly RecF proteins. *H. pylori *26695: *addB*, ATP-dependent nuclease; HP0276, hypothetical protein; *gppA*, guanosine pentaphosphate phosphohydrolase; *rfaC*, lipopolysaccharide heptosyltransferase-1. *T. aromatica*: *bcrAD*, two of the four subunits of benzoyl-CoA reductase; *orf1 *and *orf2*, hypothetical proteins. The Figure was prepared with tools available at http://cmr.jcvi.org and with the data in [20].

In *Rhodopseudomonas palustris *[19], and in the denitrifying β-*Proteobacteria*, *Thauera aromatica *[20] and *Azoarcus sp*. strain CIB [21], the *fdx *gene belongs to a cluster of genes involved in anaerobic catabolism of aromatic compounds (Figure 2). In *Thauera aromatica*, Fdx receives electrons from 2-oxoglutarate:Fdx oxidoreductase and donates them to benzoyl-CoA reductase, the ATP-dependent dearomatizing enzyme [17]. By similarity, the *fdx *gene likely belongs to a catabolic operon [16] in the other anaerobic benzoate-degrading bacteria displaying clustered homologous genes [19,21].

A case of interest is that of *Azoarcus sp*. EbN1 (called *Aromatoleum aromaticum *strain EbN1 in the most recent literature) which anaerobically degrades aromatics and displays a ferredoxin gene (improperly designated by *fxd*) in the *bcr *(benzoyl CoA reductase) genomic cluster [22]. Although it most probably binds two [4Fe-4S] clusters, the "Fxd" ferredoxin does not have the sequence characteristics of Fdx (sequence [13] of Figure 1A). Furthermore, in another part of the genome downstream of the pantetheine-phosphate adenylyltransferase gene (*coaD*), *Azoarcus sp*. EbN1 does have a *fdx *gene (locus NT01AE0820, sequence [9] of Figure 1A), potentially encoding a Fdx of the AlvinFdx family. Thus it seems unlikely that the latter Fdx participates in the anaerobic degradation of aromatics in this bacterium.

The *coaD *gene was found on the 5' side of *fdx *in several bacteria including *P. aeruginosa *PAO1. However, the involvement of Fdx in the reaction catalyzed by phosphopantetheine adenylyltransferase has not been demonstrated, and the very high-energy electrons Fdx may provide are not required in the CoA biosynthetic pathway. Thus, *coaD *and *fdx1 *do not need to be functionally linked. Furthermore, *coaD *and *fdx1 *are not always close in the sequences of many genomes, in *E. coli *K12-MG1655 for instance (Figure 2), and the layout around *fdx *is highly variable (Figure 2). In *P. aeruginosa *PAO1, a gene encoding a probable γ-glutamyltranspeptidase is located 3' of *fdx1*. This is also the case for some other strains of *P.aeruginosa *and for bacteria of the *Xanthomonas *and *Xylella *genera, but this layout is not largely conserved, even within the *Pseudomonas *genus (Figure 2). Therefore, the transcriptional characteristics of *fdx*, not belonging to *bcr *clusters, have been explored.

### Transcription of *fdx *genes encoding Alvin-like Fdxs

**Figure 3:**
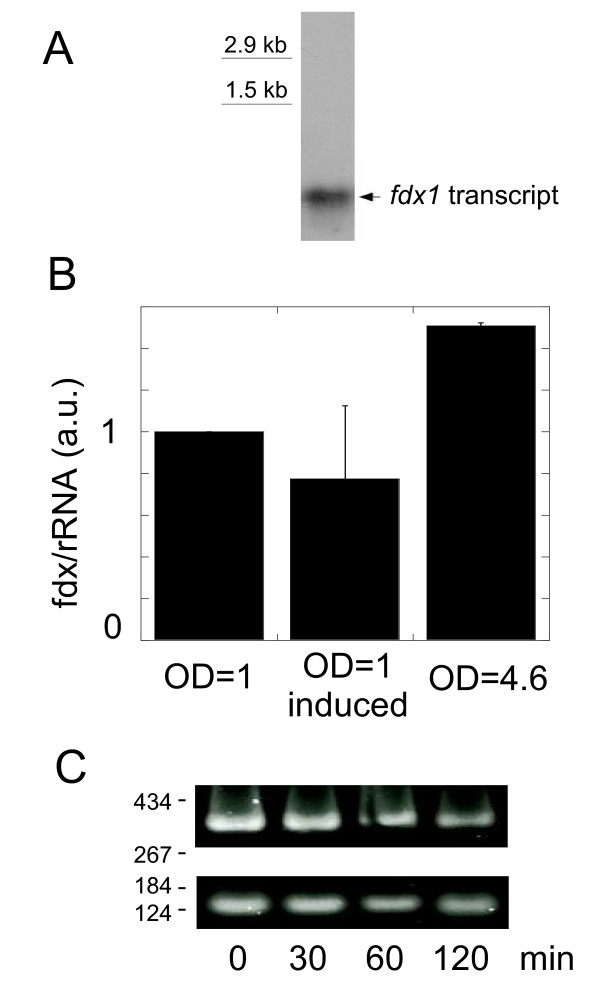
**Expression of *P. aeruginosa fdx1***. (A) For Northern blots, total RNA was hybridized to a [^32^P]-dCTP-labelled *fdx1*-probe after electrophoretic separation and the autoradiogram shown is representative of several experiments. (B) The *fdx1 *transcript was detected by RT-PCR as a 136 bp amplicon and compared to the reference 350 bp-rRNA. The ratio *fdx1*/rRNA was arbitrarily set at 1 for cells at OD = 1, and compared with induced (i.e. calcium-depleted for T3SS induction) cells, and OD = 4.6 cells. Cumulative data from 3 experiments with standard error. (C) Time course evolution of the rRNA control (upper panel) and the *fdx1 *transcript (lower panel) after OD = 1-cells had infected J774 macrophages at multiplicity of infection of 10. The time of contact with macrophages is indicated in minutes and the size scale in bp is on the left of the panels.

Northern blot analysis of *P. aeruginosa *mRNA revealed a single band of less than 500 nt hybridizing with a *fdx1 *probe (Figure 3A), both in the PAO1 and CHA strains. The small size of the *P. aeruginosa fdx1 *transcript indicates that the transcription start site must be close to the coding sequence and that it is monocistronic.

The 1 kb regions 5' of the coding sequences of the *E. coli*, *P. aeruginosa*, and *Helicobacter pylori *Fdxs do not share recognition sequences for common transcription factors. Promoter activity of the 5' sequence of *E. coli yfhL *(the *fdx *gene in this bacterium) was qualitatively reported before [23]. We also detected the *yfhL*, i.e. *fdx*, mRNA by RT-PCR (data not shown).

**Figure 4:**
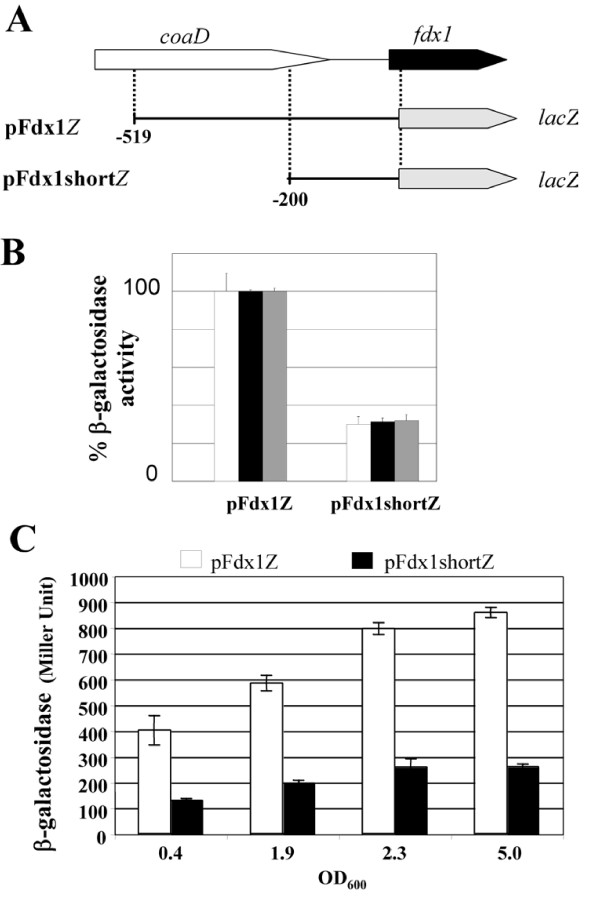
**β-Galactosidase activities in *P. aeruginosa *strains containing chromosomal *lacZ *fusions to the *fdx1 *5' sequence**. (A) Scheme of the two constructs used to monitor transcriptional activities of the *fdx1 *promoter. The -529 and -200 positions are relative to the +1 start of translation. (B) Relative β-galactosidase activities triggered by the constructs in (A) under normal conditions (white bars), for calcium depleted (for T3SS induction) cells (black bars), and for cells grown under semi-aerobic conditions with KNO_3 _(gray bars). (C) β-galactosidase activities were measured in pFdx1Z and pFdx1shortZ strains grown in LB medium at the indicated OD_600_. The reported activity values are the average of at least two independent experiments performed in duplicate or triplicate. Error bars indicate standard deviations.

To look for regulation, measurements of the *P. aeruginosa fdx1 *mRNA levels have been carried out under different conditions. It was found that the relative expression of *fdx *increased along the growth phase (Figure 3B, see also below Figure 4C). Since *P. aeruginosa *is an opportunistic pathogen, we wondered whether *fdx1 *expression was also triggered during host-bacterium interaction or co-regulated with other virulence factors. Calcium depletion by EGTA to chemically induce synthesis of the Type 3 Secretion System (T3SS) [24], a major virulence factor of *P. aeruginosa*, did not change the expression of *fdx1 *(Figure 3B). T3SS is naturally induced when bacteria contact host cells [25]. Yet, *P. aeruginosa *cells in the presence of macrophages showed similar amounts of *fdx1 *mRNA, relative to rRNA, from the time of contact up to 2 hours later (Figure 3C).

To get insight into the promoter region of the *P. aeruginosa fdx1 *gene, the fragment [519 +26] (relative to position + 1 of translation) was transcriptionally fused to the promoter-less *lacZ *gene (Figure 4). This construction, which contains a 5' truncated version of the *coaD *coding sequence, was introduced in the *attB *site of the *P. aeruginosa *CHA genome. The [519 +26] fragment was found to promote *lac*Z transcription. Transcription of *fdx1 *was independent of calcium depletion and of the presence of ExsA (data not shown), the key transcription factor of T3SS genes, in agreement with the results of RT-PCR experiments (Figure 3). Along the growth curve, β-galactosidase activity rose from 400 Miller Units at early logarithmic phase to more than 800 when reaching the stationary phase (Figure 4C), again in agreement with the results of RT-PCR experiments (Figure 3B).

Another construction with 200 bp, instead of 519 bp, upstream of the *fdx1 *coding sequence, and devoid of any *coaD *sequence, gave ca. 3 fold lower activities, indicating that the [-519 -200] region enhances transcription of *fdx1*. The number of Miller units of β-galactosidase activity also increased with the biomass under the dependence of the shortened version of the promoter region (Figure 4), as was observed with the longer one. Removing oxygen from a rich nitrate-containing medium did not change the difference between the long and shorter versions of the promoter region (Figure 4).

The carbon source (glucose or pyruvate), as well as the nitrogen one (ammonium ions or nitrate), in a minimal medium did not impact the β-galactosidase activity (data not shown). Since some Fdxs of the AlvinFdx family are involved in the degradation of aromatic compounds, *P. aeruginosa *was cultivated with 4-hydroxy benzoate as sole carbon source: in the presence of nitrate and without oxygen, *P. aeruginosa *did not grow, thus indicating that the catabolic benzoyl CoA pathway is not present in this bacterium, in agreement with the lack of benzoyl CoA reductase in the *P. aeruginosa *genome. This result excludes a single benzoyl CoA-reducing role for Fdx in all bacteria in which the *fdx *gene has been found (see above). Despite the very large number of *fdx *genes present in proteobacteria, proteins of the AlvinFdx family have only been purified from *Allochromatium vinosum *[9,11], *T. aromatica *[26], and *Azotobacter vinelandii *[27]: growth of *A. vinosum *was on synthetic medium lacking aromatic compounds [28], whereas benzoate was the unique carbon source of *T. aromatica *[20]. With oxygen as electron acceptor, *P. aeruginosa *grew on 4-hydroxy benzoate with expression of *fdx1 *at a rate similar to growth on glucose or pyruvate. This confirms that the aerobic pathway of 4-hydroxy benzoate catabolism is active in *P. aeruginosa*, but it does not require a larger *fdx1 *expression than for growth on glucose or pyruvate.

### Gene deletions

To assess the functional importance of *P. aeruginosa *Fdx, inactivation of the *fdx1 *gene was carried out. The suicide plasmid pEXΔFdx1 contained a fragment of 762 bp encompassing *fdx1 *from which the coding sequence between the sixth and the last 12 nucleotides was removed and replaced by a *Xho*I restriction site. Two other plasmids in which a Gm^*R *^cassette was cloned in both orientations, using this *Xho*I site, were also prepared. All three plasmids were introduced in the *P. aeruginosa *CHA strain by homologous recombination. The use of the cassette-less construction aimed at avoiding any polar effect triggered by the introduced DNA. Numerous attempts at excising *fdx1 *consistently afforded the wild-type genotype: this suggests that *fdx1*-deleted bacteria were selected out with this experimental strategy. Disrupting the *P. aeruginosa fdx1 *gene by directly integrating a pEX100T-based suicide plasmid into the chromosomal coding sequence (see Materials and Methods) also failed to afford viable mutants.

**Figure 5:**
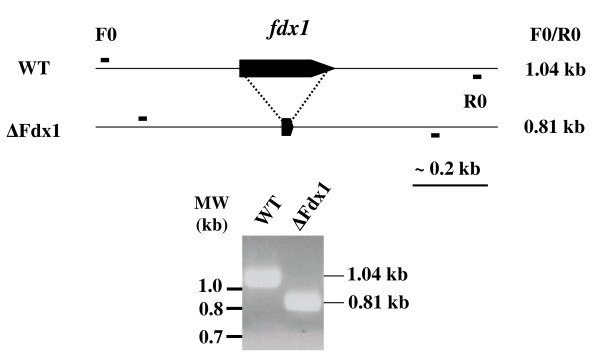
**Evidence for removal of the genomic version of P. aeruginosa fdx1**. The schematic arrangement of the genome before (WT) and after (Δ Fdx1) mutagenesis is shown above the gel with the PCR fragments amplified with the FDX-F0 and FDX-R0 primers (Table 1). The CHA cells used in this experiment contained the pVLT-FdxS plasmid with a copy of the *fdx1 *coding sequence, but without sequences complementary to the FDX-F0 and FDX-R0 primers.

Clones in which the genomic copy of *fdx1 *was deleted (Figure 5) only grew when a functional copy of the *fdx1 *gene was provided in *trans*, either on the pVLT-pFdxS plasmid (gene under its own and *pTac *promoters) or on the pJN-Fdx1 plasmid (gene under *pBAD *control), prior to integrated-plasmid counter-selection. This procedure gave around 50% of clones in which the PA0362 locus was deleted, as verified by PCR analysis. Consistently, curing the mutants of the plasmid copy of *fdx1 *did not allow us to select colonies lacking the chromosomal copy of the gene. These results indicate that the plasmids bearing *fdx1 *rescued the cells that had lost chromosomal *fdx1*, but complete lack of the gene was deleterious to *P. aeruginosa *growth. Hence this gene is essential for the *P. aeruginosa *CHA strain.

## Discussion

The fact that *fdx1 *is essential in *P. aeruginosa *challenges any speculation about its function. The present work shows that this gene is indeed expressed in *P. aeruginosa *(Figure 3), but little previous work addressed its regulation. The transcriptome subset varying between biofilm and planktonic cultures of *P. aeruginosa *PAO1 has been reported [29]: *fdx1 *transcription was increased ca. 3 times in biofilms as compared to free living bacteria. However, such variations were not confirmed in another similar study [30].

Considering other members of the AlvinFdx family, one of the two *fdx *genes in *Campylobacter jejuni *(sequence [14] of Figure 1A) was found to be iron-regulated and involved in the aerobic survival of cells in the stationary phase [31]. The sequence of another Fdx of this bacterium (sequence [7] of Figure 1A) is more similar to the Fdx consensus. We could not demonstrate iron regulation for the single *fdx *gene of *P. aeruginosa *or *E. coli *(not shown), in line with previous results obtained with *H. pylori *[32] and *P. aeruginosa *[33]. *H. pylori *strains are of particular interest since their only annotated ferredoxin gene is of the type discussed here. The encoded protein has been associated with metronidazole resistance, at least for some strains [34,35], including because it is suspected to donate electrons to a nitroreductase (the product of the *frxA *gene) that is required to activate the drug. The observation that the gene could be deleted in some, but not all, *H. pylori *strains [35] did not help assigning a function to Fdx. In particular, the actual involvement of Fdx as low potential electron shuttle between oxidoreductases in *H. pylori *as suggested [34] remains to be clearly delineated since Fdx proteins have been shown to be poor electron donors/acceptors in coupled reactions using such enzymes [36,37]. Indeed, flavodoxin has been assigned this role in *H. pylori *and *C. jejuni *[37]. Furthermore, the induced high-level expression of *frxA *resulting from the deletion of *fdx *in some *H. pylori *strains suggested a repressor function for *fdx *and additional important, but undefined, roles [35].

The genome context around the *fdx *genes is not conserved in different bacteria, and evidence for transcription as part of an operon is lacking, with the exception of clusters of genes involved in the anaerobic degradation of aromatic compounds [19-21]. In *P. aeruginosa *several, often putative, oxidoreductases can be identified in the analysis of the genome, and many low-potential electron transfer molecules coexist. *P. aeruginosa fdx1 *is transcribed as a short messenger in a constitutive-like manner, and our attempts at deleting *fdx1 *indicated that it belongs to the minority of essential genes (estimated around 10% [38]) in this bacterium. This conclusion agrees with the absence of *P. aeruginosa *transposon mutants for PA0362, both in PAO1 http://pseudomutant.pseudomonas.com/index.html and PA14 http://pga.mgh.harvard.edu/Parabiosys/projects/host-pathogen_interactions/library_construction.php libraries. An exception is in a library with *phoA *and *lacZ *insertions, in which the PA0362 locus was targeted in two mutants [38]. These insertions occur in the genomic sequence very close to the 3' end of the *fdx1 *ORF. Therefore, most of *P. aeruginosa *Fdx should be synthesized in these mutants: the variability of the C-terminus among Fdxs and inspection of the structure (Figure 1) indicate that the insertions should not completely inactivate Fdx in these mutants.

## Conclusions

The data presented herein demonstrate that donation of electrons to benzoyl-CoA reductase cannot be the sole function of ferredoxins of the AlvinFdx family. The lethality of *fdx1 *removal indicates that functional substitution of Fdx by other proteins of *P. aeruginosa *does not occur, maybe because the product of *fdx1 *fulfils other functions than conventional electron transfer between redox enzymes. This possibility was previously inferred by changes in *frxA *expression upon *fdx *removal in strains of *H. pylori *[35]. Similar suggestions arose from various kinds of data obtained with other small iron-sulfur proteins, such as thioredoxin-like ferredoxins [39] and the [2Fe-2S] *isc*-associated Fdx of *E. coli *in the secretion of cytotoxic necrotizing factor 1 [40]. Potential regulating mechanisms involving Fdx cannot be discussed at this stage, but they may include stabilization of proteins or protein complexes, electron exchange with redox-sensitive regulators, and others.

As detailed above, many bacteria of the *Proteobacteria *phylum, such as *Francisella tularensis*, *Neisseria meningitidis*, or *Yersinia pestis *among many, contain the *fdx *gene and they are human pathogens. If this gene is essential in many of them, as shown here for *P. aeruginosa*, proteins of the AlvinFdx family may provide new targets for future antibiotics.

## Methods

### Bacterial strains and growth conditions

The *P. aeruginosa *strain used in most experiments is the cystic fibrosis isolate CHA strain [41], but some experiments were also carried out with the reference PAO1 strain. *Escherichia coli *Top10 (Invitrogen) strain was used for standard cloning experiments. *P. aeruginosa *was grown on Pseudomonas Isolation Agar (PIA; Difco) plates or in liquid Luria Broth (LB) medium at 37°C with agitation, and the antibiotics used for selection on plates were carbenicillin (Cb) 500 μg/ml, tetracycline (Tc) 200 μg/ml, and gentamycin (Gm) 200 μg/ml.

For experiments aiming at measuring *fdx1 *expression under different conditions with the LacZ reporter activity, *P. aeruginosa *was diluted to an optical density of 0.1 at 600 nm (OD_600_) in the required medium. To induce the type 3 secretion system (T3SS), the *P. aeruginosa *cells were diluted in LB supplemented with 5 mM EGTA and 20 mM MgCl_2_. Control (no T3SS induction) cells were diluted in the same medium with 5 mM CaCl_2_. *P. aeruginosa *cells were grown for an additional 3 hours to a final OD of 1.0 before measurement of LacZ activity. To study the interaction with macrophages, the murine cell line J774 (ATCC) was grown in Dulbecco's modified Eagle medium (Gibco) supplemented with 10% heat-inactivated fetal calf serum (Gibco). Macrophages were seeded in 75 cm^2 ^culture flasks (BD Falcon) 20 hours before infection. *P. aeruginosa *cells were grown in LB up to an OD_600 _of 1.0. The J774 macrophages (1.8 × 10^7 ^per flask) were infected with bacteria at a multiplicity of infection of 10 for 1 or 2 hours. The supernatants were then withdrawn and the non-phagocytosed bacteria were harvested by centrifugation prior to RNA purification. In semi-aerobic growth conditions, overnight *P. aeruginosa *cultures were diluted to OD_600 _0.075 in LBN (LB with NaCl 2.5 g/L and KNO_3 _1%) into medium-filled flasks plugged with non-porous caps. The medium was saturated with N_2 _gas by bubbling for 30 min, and the cultures were grown with agitation at 37°C. To study the impact of the carbon or nitrogen source on *fdx1 *expression, *P. aeruginosa *was grown in minimal M63 medium supplemented with 0.5% casamino-acids and with either 40 mM glucose or pyruvate, or with 15 mM ammonium or 40 mM nitrate, as carbon and nitrogen sources, respectively. Growth with *p*-hydroxybenzoate as carbon source was carried out in the synthetic medium described for bacteria degrading aromatics in the absence of oxygen [42].

### Construction of *lacZ *reporter insertion

PCR amplification was used to produce the two *fdx1 *promoter fragments: primers FDX-Eco and FDX-Bam (Table 1) amplified a 555 bp fragment, and primers FDX-Eco200 (Table 1) and FDX-Bam a 237 bp fragment. The PCR products were ligated into the pCR-Blunt II-TOPO vector (Invitrogen) and sequenced. The 0.55-kb and 0.24-kb fragments were transferred into mini-CTX-*lacZ *[43], providing the pCTX-pFdx1Z and pCTX-pFdx1shortZ plasmids, respectively. The plasmids were introduced into *P. aeruginosa *by triparental conjugation, using the conjugative properties of the helper plasmid pRK2013 [44]. The transconjugants were selected on PIA plates containing tetracycline: plasmids were inserted at the chromosomal ϕCTX attachment site (*attB *site). The pFLP2 plasmid was used to excise the Flp-recombinase target cassette as described [45]. The corresponding *P. aeruginosa *strains were designated with the pFdx1Z and pFdx1shortZ extensions.

**Table 1:** Oligonucleotides used in this work.

name	Sequence (5' - 3')	comments
FDX-Eco	GAATTCGACCATGTGATCATCGCG	Construction of *lacZ *reporter
FDX-Bam	GGATCCTCAATCGTCAGTGATTTTCAGGG	Construction of *lacZ *reporter
FDX-Eco200	GAATTCgccgctctcggcggg	Construction of *lacZ *reporter
FDX-F1	GGGAAGGCGAATGTCTTCCTC	Deletion of *fdx1*
FDX-R1	GGATCAGGCCTTGCCCTC GAGGGACATCTAACAACTCC	Deletion of *fdx1*
FDX-F2	CTCGAGGGCAAGGCCTGATCCCC	Deletion of *fdx1*
FDX-R2	GGGAGTCCCGAGCCGTAAGG	Deletion of *fdx1*
FDX-F3	CCCGGGTTGTTAG**TA**GTCCCTGAAAATC	Insertion mutagenesis of *fdx1 *(inverted nucleotides in bold)
FDX-R4	CCCGGGCACTGCGGCTCGTCGTAG	Insertion mutagenesis of *fdx1*
FDX-F0	CTGGCGCACTTCGTCAAGGAG	Amplification of the genomic copy of *fdx1*
FDX-R0	GCAGAAGGAAGAATCCACCGC	Amplification of the genomic copy of *fdx1*
FDX-PstI	CCCTGCAGGTCGCGGTTGGAGTTGTTAG	Construction of pJN-Fdx1
FDX-XbaI	GGTCTAGAAGGACAGGCGCCGGCGG	Construction of pJN-Fdx1
F2PaFd	CGCAAGGCGAAGAAATCTAT	Primer for *fdx1 *RT-PCR
R2PaFd	TCCTTGCTCTCGACATTGG	Primer for *fdx1 *RT-PCR
R1bacRNA	AGAGTTTGATCCTGGCTCAG	Primer for rRNA RT-PCR
R2bacRNA	ACTGCTGCCTCCCGTAGGAG	Primer for rRNA RT-PCR

Recognition sequences for restriction enzymes are underlined

### β-Galactosidase assays

*P. aeruginosa *suspensions (0.5 ml) at an OD_600 _of 1.0 were permeabilized by addition of 20 μl of 0.1% sodium dodecyl sulfate and 20 μl of chloroform, followed by vortexing for 1 min. β-galactosidase was then assayed according to Miller [46], with up to 0.1 ml of cells, in 0.9 ml of Z buffer (Na_2_HPO_4_/NaH_2_PO_4 _0.1 M; KCl 10 mM; MgSO_4 _1 mM; 2-mercapto-ethanol 50 mM; pH 7.0) at 28°C. Reaction was initiated by addition of 0.2 ml of 4 mg/ml *o*-nitrophenyl-β-D-galactopyranoside and it was stopped with 0.5 ml of 1 M Na_2_CO_3_. OD_420 _was read after sedimentation of cell debris and the activities expressed in Miller Units [(OD_420 _× 1000)/(t_min _× Vol_ml _× OD_600_)], where t_min _is the length of the reaction in minutes.

### Deletion and insertion mutagenesis of *fdx1*

The DNA fragments needed for deletion experiments were amplified by the Splicing by Overlap Extension-Polymerase Chain Reaction (SOE-PCR). The upstream and downstream flanking regions of *fdx1 *were amplified using genomic DNA and both couples of primers, FDX-F1 and FDX-R1 (including a *Xho*I site), and FDX-F2 (including a *Xho*I site) and FDX-R2 (Table 1). Each of the two fragments of 387 bp and 396 bp, respectively, were used as template for a third PCR step using primers FDX-F1 and FDX-R2. The resulting 762 bp fragment was cloned into pCR-Blunt II-TOPO vector (Invitrogen) and sequenced: the *fdx1 *coding sequence between the sixth and the last 12 nucleotides was thus removed and replaced by a *Xho*I restriction site. After cleavage with *Eco*RI and treatment with the Klenow fragment of DNA polymerase I, the SOE-PCR fragment was inserted into the suicide plasmid pEX-100T [47] cut by *Sma*I, giving the pEXΔFdx1 plasmid. Of note, this plasmid contains the counter-selectable *sacB *marker from *Bacillus subtilis*, which confers sensitivity to sucrose. A 856 bp fragment, corresponding to the Gm resistance cassette, was excised from pUCGm [48] by *Sma*I, and cloned in both orientation into pEXΔFdx1 cut with *Xho*I and treated with the Klenow fragment of DNA polymerase I: this gave the pEXΔFdx1GmS and pEXΔFdx1GmAS plasmids. The three pEX100T-derived plasmids were introduced into the *P. aeruginosa *CHA strain using triparental conjugation. Co-integration events were selected on PIA plates containing Cb (pEXΔFdx1), or Cb and Gm (pEXΔFdx1GmS/AS). Insertion of the plasmid was verified by PCR using the appropriate pairs of primers. Single colonies were then plated on PIA medium containing 5% sucrose to select for the loss of plasmid: the resulting strains were checked for Cb sensitivity, for Gm resistance when required, and for *fdx1 *(wild-type or deleted gene) genotype by PCR.

For insertion mutagenesis, a 5' fragment of *fdx1 *was amplified by PCR using primers FDX-F3 and FDX-R4 (Table 1) containing *Sma*I sites at the 5' end: in the resulting 167 bp fragment, the ATG codon was replaced by a TGA stop codon in the FDX-F3 primer. This fragment was cloned into pCR-Blunt II-TOPO vector and sequenced. After *Sma*I hydrolysis, the fragment was cloned into the suicide plasmid pEX100T cut with the same enzyme, yielding plasmid pEXΔFdxF3R4. This plasmid was introduced by triparental conjugation into the CHA strain and the cointegration event was selected on PIA plates with Cb.

For experiments in which deletion mutants were rescued by a wild-type copy of *fdx1*, two plasmids, pVLT-FdxS and pJN-Fdx1, were constructed and transformed into the *P. aeruginosa *co-integration strains prior to *sacB *counter-selection. To assemble pVLT-FdxS, a 1.06-kb genomic fragment was amplified using primers FDX-F1 and FDX-R2, cloned into pCR-Blunt II-TOPO vector, and sequenced. The fragment contained the entire PA0362 ORF (*fdx*) and 361 bp upstream of the starting codon. After hydrolysis with *Eco*RI and treatment with the Klenow fragment of DNA polymerase I, the PCR fragment was inserted into the replicative plasmid pVLT31 [49] cut by *Sma*I, in the same transcriptional orientation as that of *pTac*, leading to pVLT-FdxS (Tc resistance). To construct pJN-Fdx1, a 308 bp fragment encompassing PA0362 was amplified using primers FDX-PstI and FDX-XbaI (Table 1), cloned into pCR-Blunt II-TOPO vector, and sequenced. The fragment was hydrolyzed by *Pst*I and *Xba*I and cloned into the replicative plasmid pJN105 [50] cut with the same enzymes. This gave the pJN-Fdx1 plasmid in which the *fdx1 *gene is under the control of *pBAD *(Gm^r^). The co-integration strains were transformed with the pVLT-FdxS or pJN-Fdx1 plasmids and grown on PIA-Sucrose 5%-Tc or PIA-Sucrose 5%-Gm-Arabinose 2%, respectively. The selected Suc^R ^et Cb^S ^clones were analyzed by PCR as in Figure 5.

### Northern Blots and RT-PCR

To study expression of the *fdx *genes, total RNA from harvested bacteria was extracted with the Trizol reagent (Invitrogen, Carlsbad, CA, USA). Absence of co-purified genomic DNA was assessed by PCR reactions using 100 ng of extracted RNA as template: the absence of any amplified band was taken as evidence for removal of contaminating DNA. Northern blot analysis was performed using the glyoxal method [51]. Equal RNA loading (~5-10 μg) was based on both optical density measurements and estimates of the amounts of rRNA [51]. [^32^P]-dCTP-labeled, *fdx1*-specific, DNA probe was prepared by random hexanucleotide-primed synthesis. [^32^P]-dCTP (3000 Ci mmol^-1^) was purchased from the Institute of Radioisotopes & Radiodiagnostic Products, NCSR Demokritos, Athens, Greece.

In RT-PCR experiments, complementary DNA synthesis was carried out with an engineered version of the Moloney Murine Leukemia Virus reverse transcriptase provided in the RevertAid™ H Minus First Strand cDNA Synthesis Kit (Fermentas, St Leon-Rot, Germany) and a random hexamer primer. Calibration of the PCR amplification step was done by first using a range of template cDNA over a varying number of cycles with primers targeting either the *fdx *transcript of interest or rRNA as a reference transcript. Comparison between samples was then obtained by loading non-saturating amplified DNA on 3.5% agarose gels.

### Computational tools

Sequence comparisons were performed with various versions of the Blast program at NCBI http://blast.ncbi.nlm.nih.gov/Blast.cgi. Genome searching made use of the tools available at the Comprehensive Microbial Resource web site (Data Release 21.0 at http://cmr.jcvi.org/tigr-scripts/CMR/CmrHomePage.cgi. The AlvinFdx family was defined by the 6-8 amino acids insertion between two cysteine ligands of cluster II and the C-terminal piece of ca. 20-40 amino acids following the cluster-binding domain (Figure 1).

## Abbreviations

Fdx: ferredoxin; Alvin: short-hand for *Allochromatium vinosum*; *fdx1*, gene of the *Pseudomonas aeruginosa *PA0362 locus; *fdx*, gene encoding a Fdx similar to that of AlvinFdx in other bacteria than *Pseudomonas aeruginosa*; SOE-PCR: Splicing by Overlap Extension-Polymerase Chain Reaction; T3SS: type 3 secretion system; Cb: carbenicillin; Tc: tetracycline; Gm: gentamycin; PIA: Pseudomonas Isolation Agar.

## Authors' contributions

SE participated in the design of the study, carried out the molecular genetic experiments, interpreted the data and corrected the manuscript. GE carried out some RT-PCR experiments. PP carried out the Northern-Blot and some RT-PCR experiments. GD participated in setting up the Northern-Blot experiments, interpreted the data and corrected the manuscript. PK participated in the design of the study, sought financial support, participated in setting up experiments and corrected the manuscript. JMM designed and coordinated the study, sought financial support, participated in setting up experiments, performed database queries, interpreted data, and wrote the manuscript. All authors read and approved the final manuscript.

## References

[B1] Meyer J Iron-sulfur protein folds, iron-sulfur chemistry, and evolution J Biol Inorg Chem 2008 13 2 157 170 10.1007/s00775-007-0318-7 17992543

[B2] Andreini C Banci L Bertini I Elmi S Rosato A Non-heme iron through the three domains of life Proteins 2007 67 2 317 324 10.1002/prot.21324 17286284

[B3] Mortenson LE Valentine RC Carnahan JE An electron transport factor from *Clostridium pasteurianum* Biochem Biophys Res Commun 1962 7 448 452 10.1016/0006-291X(62)90333-9 14476372

[B4] Meyer J Ferredoxins of the third kind FEBS Lett 2001 509 1 1 5 10.1016/S0014-5793(01)03049-6 11734195

[B5] Meyer J Miraculous catch of iron-sulfur protein sequences in the Sargasso Sea FEBS Lett 2004 570 1-3 1 6 10.1016/j.febslet.2004.06.030 15251429

[B6] Schönheit P Brandis A Thauer RK Ferredoxin degradation in growing *Clostridium pasteurianum *during periods of iron deprivation Arch Microbiol 1979 120 1 73 76 10.1007/BF00413277 426601

[B7] La Roche J Boyd PW McKay RML Geider RJ Flavodoxin as an in situ marker for iron stress in phytoplankton Nature 1996 382 6594 802 804 10.1038/382802a0

[B8] Stover CK Pham XQ Erwin AL Mizoguchi SD Warrener P Hickey MJ Brinkman FS Hufnagle WO Kowalik DJ Lagrou M Complete genome sequence of *Pseudomonas aeruginosa *PA01, an opportunistic pathogen Nature 2000 406 6799 959 964 10.1038/35023079 10984043

[B9] Moulis J-M Molecular cloning and expression of the gene encoding *Chromatium vinosum *2[4Fe-4S] ferredoxin Biochim Biophys Acta 1996 1308 1 12 14 8765743 10.1016/0167-4781(96)00082-6

[B10] Giastas P Pinotsis N Efthymiou G Wilmanns M Kyritsis P Moulis J-M Mavridis IM The structure of the 2[4Fe-4S] ferredoxin from *Pseudomonas aeruginosa *at 1.32-Å resolution: comparison with other high-resolution structures of ferredoxins and contributing structural features to reduction potential values J Biol Inorg Chem 2006 11 4 445 458 10.1007/s00775-006-0094-9 16596388

[B11] Bachofen R Arnon DI Crystalline ferredoxin from the photosynthetic bacterium *Chromatium* Biochim Biophys Acta 1966 120 2 259 265 10.1016/0926-6585(66)90345-1 4381411

[B12] Kyritsis P Hatzfeld OM Link TA Moulis J-M The two [4Fe-4S] clusters in *Chromatium vinosum *ferredoxin have largely different reduction potentials. Structural origin and functional consequences J Biol Chem 1998 273 25 15404 15411 10.1074/jbc.273.25.15404 9624123

[B13] Kyritsis P Kümmerle R Huber JG Gaillard J Guigliarelli B Popescu C Münck E Moulis J-M Unusual NMR, EPR, and Mössbauer properties of *Chromatium vinosum *2[4Fe-4S] ferredoxin Biochemistry 1999 38 19 6335 6345 10.1021/bi982894u 10320364

[B14] Moulis J-M Sieker LC Wilson KS Dauter Z Crystal structure of the 2[4Fe-4S] ferredoxin from *Chromatium vinosum*: evolutionary and mechanistic inferences for [3/4Fe-4S] ferredoxins Protein Sci 1996 5 9 1765 1775 10.1002/pro.5560050902 8880900 PMC2143546

[B15] Saridakis E Giastas P Efthymiou G Thoma V Moulis J-M Kyritsis P Mavridis IM Insight into the protein and solvent contributions to the reduction potentials of [4Fe-4S]^(2+/+)^clusters: crystal structures of the *Allochromatium vinosum *ferredoxin variants C57A and V13G and the homologous *Escherichia coli *ferredoxin J Biol Inorg Chem 2009 14 5 783 799 10.1007/s00775-009-0492-x 19290553

[B16] Fuchs G Anaerobic metabolism of aromatic compounds Ann N Y Acad Sci 2008 1125 82 99 10.1196/annals.1419.010 18378589

[B17] Dörner E Boll M Properties of 2-oxoglutarate:ferredoxin oxidoreductase from *Thauera aromatica *and its role in enzymatic reduction of the aromatic ring J Bacteriol 2002 184 14 3975 3983 10.1128/JB.184.14.3975-3983.2002 12081970 PMC135165

[B18] Boll M Fuchs G Tilley G Armstrong FA Lowe DJ Unusual spectroscopic and electrochemical properties of the 2[4Fe-4S] ferredoxin of *Thauera aromatica* Biochemistry 2000 39 16 4929 4938 10.1021/bi9927890 10769152

[B19] Egland PG Pelletier DA Dispensa M Gibson J Harwood CS A cluster of bacterial genes for anaerobic benzene ring biodegradation Proc Natl Acad Sci USA 1997 94 12 6484 6489 10.1073/pnas.94.12.6484 9177244 PMC21076

[B20] Breese K Boll M Alt-Mörbe J Schägger H Fuchs G Genes coding for the benzoyl-CoA pathway of anaerobic aromatic metabolism in the bacterium *Thauera aromatica* Eur J Biochem 1998 256 1 148 154 10.1046/j.1432-1327.1998.2560148.x 9746358

[B21] López Barragán MJ Carmona M Zamarro MT Thiele B Boll M Fuchs G García JL Díaz E The *bzd *gene cluster, coding for anaerobic benzoate catabolism, in *Azoarcus *sp. strain CIB J Bacteriol 2004 186 17 5762 5774 10.1128/JB.186.17.5762-5774.2004 15317781 PMC516837

[B22] Rabus R Kube M Heider J Beck A Heitmann K Widdel F Reinhardt R The genome sequence of an anaerobic aromatic-degrading denitrifying bacterium, strain EbN1 Arch Microbiol 2005 183 1 27 36 10.1007/s00203-004-0742-9 15551059

[B23] Zaslaver A Bren A Ronen M Itzkovitz S Kikoin I Shavit S Liebermeister W Surette MG Alon U A comprehensive library of fluorescent transcriptional reporters for *Escherichia coli* Nat Methods 2006 3 8 623 628 10.1038/nmeth895 16862137

[B24] Frank DW The exoenzyme S regulon of *Pseudomonas aeruginosa* Mol Microbiol 1997 26 4 621 629 10.1046/j.1365-2958.1997.6251991.x 9427393

[B25] Vallis AJ Yahr TL Barbieri JT Frank DW Regulation of ExoS production and secretion by *Pseudomonas aeruginosa *in response to tissue culture conditions Infect Immun 1999 67 2 914 920 9916108 10.1128/iai.67.2.914-920.1999 PMC96404

[B26] Boll M Fuchs G Identification and characterization of the natural electron donor ferredoxin and of FAD as a possible prosthetic group of benzoyl-CoA reductase (dearomatizing), a key enzyme of anaerobic aromatic metabolism Eur J Biochem 1998 251 3 946 954 10.1046/j.1432-1327.1998.2510946.x 9490071

[B27] Gao-Sheridan HS Pershad HR Armstrong FA Burgess BK Discovery of a novel ferredoxin from *Azotobacter vinelandii *containing two [4Fe-4S] clusters with widely differing and very negative reduction potentials J Biol Chem 1998 273 10 5514 5519 10.1074/jbc.273.10.5514 9488675

[B28] Huber JG Gaillard J Moulis J-M NMR of *Chromatium vinosum *ferredoxin: evidence for structural inequivalence and impeded electron transfer between the two [4Fe-4S] clusters Biochemistry 1995 34 1 194 205 10.1021/bi00001a024 7819196

[B29] Whiteley M Bangera MG Bumgarner RE Parsek MR Teitzel GM Lory S Greenberg EP Gene expression in *Pseudomonas aeruginosa *biofilms Nature 2001 413 6858 860 864 10.1038/35101627 11677611

[B30] Waite RD Paccanaro A Papakonstantinopoulou A Hurst JM Saqi M Littler E Curtis MA Clustering of *Pseudomonas aeruginosa *transcriptomes from planktonic cultures, developing and mature biofilms reveals distinct expression profiles BMC Genomics 2006 7 162 10.1186/1471-2164-7-162 16800888 PMC1525188

[B31] van Vliet AH Baillon MA Penn CW Ketley JM The iron-induced ferredoxin FdxA of *Campylobacter jejuni *is involved in aerotolerance FEMS Microbiol Lett 2001 196 2 189 193 10.1016/S0378-1097(01)00067-2 11267778

[B32] Ernst FD Bereswill S Waidner B Stoof J Mader U Kusters JG Kuipers EJ Kist M van Vliet AH Homuth G Transcriptional profiling of *Helicobacter pylori *Fur- and iron-regulated gene expression Microbiology 2005 151 Pt 2 533 546 10.1099/mic.0.27404-0 15699202

[B33] Palma M Worgall S Quadri LE Transcriptome analysis of the *Pseudomonas aeruginosa *response to iron Arch Microbiol 2003 180 5 374 379 10.1007/s00203-003-0602-z 14513207

[B34] Kaakoush NO Asencio C Mégraud F Mendz GL A redox basis for metronidazole resistance in *Helicobacter pylori* Antimicrob Agents Chemother 2009 53 5 1884 1891 10.1128/AAC.01449-08 19223619 PMC2681531

[B35] Mukhopadhyay AK Jeong JY Dailidiene D Hoffman PS Berg DE The *fdxA *ferredoxin gene can down-regulate *frxA *nitroreductase gene expression and is essential in many strains of *Helicobacter pylori* J Bacteriol 2003 185 9 2927 2935 10.1128/JB.185.9.2927-2935.2003 12700272 PMC154416

[B36] Moulis J-M Davasse V Probing the role of electrostatic forces in the interaction of *Clostridium pasteurianum *ferredoxin with its redox partners Biochemistry 1995 34 51 16781 16788 10.1021/bi00051a028 8527453

[B37] St Maurice M Cremades N Croxen MA Sisson G Sancho J Hoffman PS Flavodoxin:quinone reductase (FqrB): a redox partner of pyruvate:ferredoxin oxidoreductase that reversibly couples pyruvate oxidation to NADPH production in *Helicobacter pylori *and *Campylobacter jejuni* J Bacteriol 2007 189 13 4764 4773 10.1128/JB.00287-07 17468253 PMC1913460

[B38] Jacobs MA Alwood A Thaipisuttikul I Spencer D Haugen E Ernst S Will O Kaul R Raymond C Levy R Comprehensive transposon mutant library of *Pseudomonas aeruginosa* Proc Natl Acad Sci USA 2003 100 24 14339 14344 10.1073/pnas.2036282100 14617778 PMC283593

[B39] Meyer J Andrade SLA Einsle O Messerschmidt A Thioredoxin-like [2Fe-2S] ferredoxin Handbook of Metalloproteins 2008 John Wiley & Sons, Ltd

[B40] Yu H Kim KS Ferredoxin is involved in secretion of cytotoxic necrotizing factor 1 across the cytoplasmic membrane in *Escherichia coli *K1 Infect Immun 2010 78 2 838 844 10.1128/IAI.00674-09 19917710 PMC2812213

[B41] Toussaint B Delic-Attree I Vignais PM *Pseudomonas aeruginosa *contains an IHF-like protein that binds to the *algD *promoter Biochem Biophys Res Commun 1993 196 1 416 421 10.1006/bbrc.1993.2265 8216322

[B42] Tschech A Fuchs G Anaerobic degradation of phenol by pure cultures of newly isolated denitrifying pseudomonads Arch Microbiol 1987 148 3 213 217 10.1007/BF00414814 3675113

[B43] Becher A Schweizer HP Integration-proficient *Pseudomonas aeruginosa *vectors for isolation of single-copy chromosomal *lacZ *and *lux *gene fusions Biotechniques 2000 29 5 948 950 952 11084852 10.2144/00295bm04

[B44] Figurski DH Helinski DR Replication of an origin-containing derivative of plasmid RK2 dependent on a plasmid function provided in *trans* Proc Natl Acad Sci USA 1979 76 4 1648 1652 10.1073/pnas.76.4.1648 377280 PMC383447

[B45] Hoang TT Karkhoff-Schweizer RR Kutchma AJ Schweizer HP A broad-host-range Flp-FRT recombination system for site-specific excision of chromosomally-located DNA sequences: application for isolation of unmarked *Pseudomonas aeruginosa *mutants Gene 1998 212 1 77 86 10.1016/S0378-1119(98)00130-9 9661666

[B46] Miller JH Experiments in molecular genetics 1972 Cold Spring Harbor, New York: Cold Spring Harbor Laboratory Press

[B47] Schweizer HP Hoang TT An improved system for gene replacement and *xylE *fusion analysis in *Pseudomonas aeruginosa* Gene 1995 158 1 15 22 10.1016/0378-1119(95)00055-B 7789804

[B48] Schweizer HP Small broad-host-range gentamycin resistance gene cassettes for site-specific insertion and deletion mutagenesis Biotechniques 1993 15 831 833 8267974

[B49] de Lorenzo V Eltis L Kessler B Timmis KN Analysis of *Pseudomonas *gene products using *lacIq/Ptrp-lac *plasmids and transposons that confer conditional phenotypes Gene 1993 123 1 17 24 10.1016/0378-1119(93)90533-9 8380783

[B50] Newman JR Fuqua C Broad-host-range expression vectors that carry the L-arabinose-inducible *Escherichia coli **araBAD *promoter and the *araC *regulator Gene 1999 227 2 197 203 10.1016/S0378-1119(98)00601-5 10023058

[B51] Sambrook J Fritsch EF Maniatis T Molecular Cloning: A Laboratory Manual 1989 2 Cold Spring Harbor, NY, USA: Cold Spring Harbor Laboratory Press

